# Moss BRCA2 lacking the canonical DNA-binding domain promotes homologous recombination and binds to DNA

**DOI:** 10.1093/nar/gkaf856

**Published:** 2025-09-05

**Authors:** Alice Chanteau, Suliane Quilleré, Arthur Crouset, Sreejith Allipra, Ulysse Tuquoi, Pierre-François Perroud, Simona Miron, Pauline Dupaigne, Sophie Zinn-Justin, Fabien Nogué, Rajeev Kumar

**Affiliations:** Université Paris-Saclay, INRAE, AgroParisTech, Institut Jean-Pierre Bourgin for Plant Sciences (IJPB), 78000 Versailles, France; Institute for Integrative Biology of the Cell (I2BC), CEA, CNRS UMR 9198, Université Paris-Saclay, 91190 Gif-sur-Yvette, France; Université Paris-Saclay, INRAE, AgroParisTech, Institut Jean-Pierre Bourgin for Plant Sciences (IJPB), 78000 Versailles, France; Université Paris-Saclay, INRAE, AgroParisTech, Institut Jean-Pierre Bourgin for Plant Sciences (IJPB), 78000 Versailles, France; Université Paris-Saclay, INRAE, AgroParisTech, Institut Jean-Pierre Bourgin for Plant Sciences (IJPB), 78000 Versailles, France; Université Paris-Saclay, INRAE, AgroParisTech, Institut Jean-Pierre Bourgin for Plant Sciences (IJPB), 78000 Versailles, France; Institute for Integrative Biology of the Cell (I2BC), CEA, CNRS UMR 9198, Université Paris-Saclay, 91190 Gif-sur-Yvette, France; Genome Maintenance and Molecular Microscopy, UMR9019 CNRS, Université Paris-Saclay, 94805 Gustave Roussy, Villejuif, France; Institute for Integrative Biology of the Cell (I2BC), CEA, CNRS UMR 9198, Université Paris-Saclay, 91190 Gif-sur-Yvette, France; Université Paris-Saclay, INRAE, AgroParisTech, Institut Jean-Pierre Bourgin for Plant Sciences (IJPB), 78000 Versailles, France; Université Paris-Saclay, INRAE, AgroParisTech, Institut Jean-Pierre Bourgin for Plant Sciences (IJPB), 78000 Versailles, France

## Abstract

BRCA2 is crucial for mediating homology-directed DNA repair (HDR) through its binding to single-stranded DNA (ssDNA) and the recombinases RAD51 and DMC1. Most BRCA2 orthologs have a canonical DNA-binding domain (DBD) with the exception of *Drosophila melanogaster*. It remains unclear whether such a noncanonical BRCA2 variant without DBD possesses a DNA-binding activity. Here, we identify a new noncanonical BRCA2 in the model plant *Physcomitrium patens* (PpBRCA2). We establish that PpBRCA2 is essential for genome integrity maintenance, somatic DNA double-strand break (DSB) repair, HDR-mediated gene targeting, and RAD51 foci recruitment at DNA break sites. PpBRCA2 is also critical for DSB repair during meiosis. Interestingly, PpBRCA2 interacts strongly with RAD51 but weakly with DMC1, suggesting a distinct meiotic function compared to other BRCA2 homologs. Despite lacking the canonical DBD, PpBRCA2 binds ssDNA through its disordered N-terminal region and efficiently promotes HDR. Our work highlights that the ssDNA binding capacity of BRCA2 homologs is conserved regardless of the presence of a canonical DBD and provides a deeper understanding of BRCA2’s functional diversity across species.

## Graphical Abstract

**Graphical Abstract:**
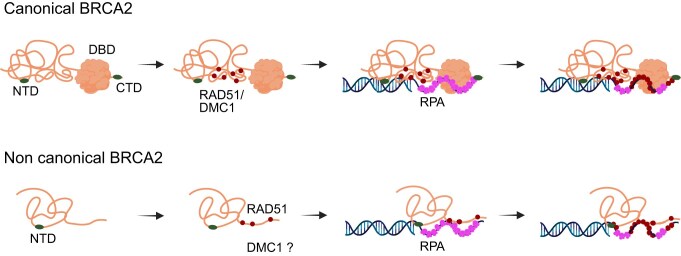

## Introduction

The BREAST CANCER gene 2 (BRCA2) preserves genomic integrity in most eukaryotes. In humans, mutations in the *BRCA2 gene* predispose to a high risk of developing breast, ovarian, and other types of cancers [1]. The loss of BRCA2 functions in various eukaryotic species exhibits a spectrum of genome instability traits, including hypersensitivity to genotoxic agents, chromosomal breakage, and aberrations [2–9]. BRCA2 plays diverse roles in genome maintenance, including protecting stalled replication forks, suppressing single-stranded DNA (ssDNA) gaps, repairing interstrand DNA crosslinks, and cytotoxic DNA double-strand breaks (DSBs) [10–14]. BRCA2 is essential for the repair of accidental breaks or programmed DSBs during meiosis through homologous recombination, also called homology-directed DNA repair (HDR). Alternatively, other pathways including nonhomologous end joining (NHEJ) can repair DSB [15].

BRCA2 is a crucial mediator of HDR in somatic and meiotic cells. During HDR, Replication Protein A (RPA) binds to 3’ ssDNA overhangs generated by nucleolytic processing of DSBs. Two eukaryotic structurally and functionally related recombinases—RAD51 and its meiosis-specific paralog DMC1—replace RPA to form nucleoprotein filaments [16, 17]. Only RAD51 acts in somatic cells, while both RAD51 and DMC1 are necessary during meiosis. These nucleofilaments drive homology searches and strand invasion into an intact donor template, producing recombination intermediates that are subsequently resolved as crossover (CO) or noncrossover repair products [16]. However, under physiological conditions, RAD51 and DMC1 cannot outcompete RPA for ssDNA due to RPA’s higher binding affinity [18, 19] and require various mediator proteins that facilitate the formation and activation of RAD51 and DMC1 nucleofilaments [20]. BRCA2 nucleates and stabilizes recombinase nucleofilaments through its direct interaction with recombinases and binding to DNA [21–24]. With its partner DSS1, BRCA2 facilitates RPA displacement from ssDNA, enabling nucleoprotein filament assembly [19, 25]. DSS1 attenuates RPA’s affinity for ssDNA through DNA mimicry [19]. Consequently, BRCA2 and DSS1 are essential for forming RAD51 and DMC1 nuclear foci during HDR [3, 7, 26]. RAD52 also interacts with DSS1 and is synthetically lethal with BRCA2, suggesting RAD52 can support HDR in BRCA2-deficient cells [27, 28].

BRCA2 interacts with RAD51 and DMC1 through highly conserved BRC repeats (∼35 amino acids) found in one or multiple copies across BRCA2 homologs [29]. Human BRCA2 contains eight BRC repeats, each with a varying binding affinity to RAD51 and DMC1 [30, 31]. Each human BRC repeat has two conserved key tetrameric hydrophobic motifs that interact with monomeric RAD51: (i) the FxxA motif, which mimics the RAD51 oligomerization interface and interacts with its catalytic domain [32]; (ii) an φφx[E/D] motif (φ = hydrophobic residue), which engages a different hydrophobic pocket of the RAD51’s catalytic domain [32]. These two distinct motifs have stimulating and inhibiting effects on nucleoprotein filaments *in vitro*, providing insights into how BRC repeats can regulate RAD51 oligomerization [32]. Additionally, BRCA2 regions outside BRC repeats, such as human BRCA2 C-terminal Recombinase Binding (CTRB) and PhePP motifs, can engage RAD51 and DMC1 nucleoprotein filaments, respectively [21, 24, 33, 34].

Most BRCA2 homologs contain a well-folded canonical ssDNA-binding domain (DBD) and additional auxiliary regions for ssDNA and/or double-stranded DNA (dsDNA) interaction [29, 34–36]. They together collectively facilitate nucleofilament formation through direct binding to ssDNA or via a diffusion-assisted delivery mechanism [37]. The canonical DBD is a well-structured and highly conserved region composed of a helical domain, three oligonucleotide-binding (OB) folds, and a tower domain [25]. It harbors most known cancer-associated pathogenic mutations [38]. The small acidic protein DSS1 binds to the canonical DBD in humans [25, 39] and restrains the dsDNA binding activity of the DBD to ensure BRCA2 targeting to ssDNA [40]. Notably, the *Drosophila melanogaster* BRCA2 homolog (DmBRCA2) lacks the canonical DBD [2, 4]. Drosophila BRCA2 forms foci at DSB sites and recruits RAD51 to mediate mitotic and meiotic DNA repair, but it remains unclear whether DmBRCA2 binds to DNA at all.

While a flowering model plant *Arabidopsis thaliana* has two BRCA2 homologs (AtBRCA2A and AtBRCA2B) containing four BRC repeats and a canonical DBD [7], no BRCA2 homolog was identified in moss genomes so far [41]. *Physcomitrium patens* is a moss model plant from the bryophyte clade and is often called a “green yeast” due to its high gene-targeting (GT) efficiency, comparable to *Saccharomyces cerevisiae*, allowing precise genetic modifications via homologous recombination [42]. Over the past few decades, *P. patens* has emerged as a powerful model for studying HDR, DNA repair, and genome editing [43]. Here, we report the identification and functional characterization of the *P. patens* BRCA2 homolog (PpBRCA2). The PpBRCA2 contains BRC repeats but lacks the canonical DBD, as previously observed for *D. melanogaster* BRCA2. We have delineated the mechanism of noncanonical PpBRCA2 in HDR, DSB repair, and genome stability, demonstrating its mediator role and its DNA-binding activity despite the absence of a canonical DBD. Based on our findings, we propose that two classes of BRCA2 homologs exist in eukaryotes: canonical BRCA2 with DBD and noncanonical BRCA2 lacking DBD.

## Materials and methods

### Plant material and culture

The *P. patens* accessions “Gransden” and “Reute” were used as wild-type (WT) references [44]. Four *BRCA2* full deletion lines were generated using the CRISPR-Cas9 system with two guide RNAs targeting the start codon and 3′ untranslated region (UTR) of the Pp6C10_10830/*PpBRCA2* gene: *brca2Δ-1* and *brca2Δ-2* in the Reute ecotype, and *brca2Δ-3* and *brca2Δ-4* in the Gransden ecotype. The *rad51-1-1rad51-2-1* and *rad51-1-2rad51-2-2* double mutants were generated by targeting *PpRAD51-1* (*Pp6c11_11820*) and *PpRAD51-2* (*Pp6c7_5100*) genes by the CRISPR-Cas9 system in the Reute background only. All guides and genotyping primers are listed in the supplementary DATASET_S3.

Moss strains were maintained and propagated on rich agar PpNH4 medium overlaid with cellophane or as spot inocula on minimal BCD medium for growth and sporogenesis assays [45, 46]. Culture chamber conditions were set at 60% humidity with 16/8 h light/dark long-day photoperiod at 23°C. Protoplast isolation, polyethylene glycol (PEG)-mediated transfection, and selection of transformants followed established protocols [47]. The short-day photoperiod of 8/16h light/dark at 15°C was used for sporogenesis induction on the BCD medium [45]. Young sporophytes fixed in ethanol-acetic acid (3:1) were analyzed for meiosis [48] and mature sporophytes were assessed for spore viability by staining with or without Alexander solution [49] and for spore germination rates [50]. Statistical analyses and graphs were generated using Prism GraphPad 10.3.0.

### Genes identification

For moss BRCA2 identification, HHpred searches with full-length *A. thaliana* BRCA2A (At4g00020) were performed as described previously [51, 52]. The *Pp6C10_10830* gene encoding PpBRCA2 without intron was cloned using *P. patens* genomic DNA with polymerase chain reaction (PCR) primers listed in DATASET_S3. The PSI-BLAST searches using *A. thaliana* BRCA2A as a query and the Phytozome.v13 database identified BRCA2 orthologs from the following species: *Ceratopteris richardii* (Ceric.07G096700), *Marchantia polymorpha* (Mapoly0045s0096), *Micromonas pusilla* (MicpuC2.est_orfs.10_1849_4269375:2), and *Chlamydomonas reinhardtii* (Cre13.g566900_4532). *Anthoceros agrestis* BRCA2 (AagrOXF_EVM_utg000116l.287) was identified using a specific Anthoceros BLAST database [53] (https://www.hornworts.uzh.ch/en/Blast.html). Protein sequence alignments including BRC repeats of HsBRCA2 (HGNC:1101) and DmBRCA2 (FB: FBgn0050169) were performed using CLUSTAL Omega [54].

### Vector design, cloning, and gene expression

All PCR primers and single guide RNA (sgRNAs) are listed in DATASET_S3. The guide RNAs targeting *PpBRCA2* (*Pp6c10_10830*), *PpRAD51-1* (*Pp6c11_11820*), or *PpRAD51-2* (*Pp6c7_5100*) genes were designed using the CRISPOR 4.97 tool (https://crispor.gi.ucsc.edu/) and were cloned into sgRNA expression cassettes either in the pDONR207 background by Gateway^TM^ (Thermo Fisher Scientific) or in the pDGB3 background by GoldenBraid cloning [55, 56]. For GFP fusion at the PpBRCA2 N-terminal, a donor matrix harboring the *eGFP* encoding sequence flanked by *PpBRCA2* 5’ UTR and coding DNA sequence (CDS) was synthesized by Twist Bioscience (San Francisco, CA, USA) and cloned into a pDONR207-NeoR vector by Gateway^TM^ BP reaction (Thermo Fisher Scientific). *PpBRCA2* guide RNA targeting the start codon was used for GFP insertion at the endogenous locus. The complementation studies were performed with the synthesized CDS of PpBRCA2* with polymorphisms, *CpBRCA2, AtBRCA2*, and *AtBRCA2^ΔDBD^* without DBD flanked with 5’ and 3’ UTR at the *PpBRCA2* locus and cloned into a pTwistAmpHighCopy plasmid by Twist Bioscience (San Francisco, CA, USA). The CDS encoding *PpBRCA2* was cloned in the pUbi-Gateway vector for transient expression in *P. patens* [50, 57]. Through CRISPR-Cas9 and a sgRNA targeting the deleted *PpBRCA2* locus, DSB was generated to facilitate the insertion of PpBRCA2*, CpBRCA2, or *AtBRCA2^ΔDBD^* from the above-cited repair matrices, while transiently expressing *PpBRCA2*. Plants with insertions were allowed to grow without selection and the absence of pUbi-PpBRCA2 integration was monitored by PCR. For yeast two hybrid (Y2H) assays, DNA sequences encoding *PpBRCA2*, *PpRAD51-1*, *PpRAD51-2*, *PpDMC1*, or *BRC* repeats were cloned in pGAD-T7 and pGBK-T7 vectors. The fidelity of all constructs was confirmed by sequencing. For reverse transcriptase-polymerase chain reaction (RT-PCR), total RNA was extracted from protonema using the RNeasy Plant mini kit (Qiagen, cat. No. 74904). The complementary DNA (cDNA) synthesis was performed with 1 mg of total RNA from respective genotypes using the RevertAid cDNA Synthesis Kit (K1622; Thermo Fisher Scientific, Waltham, MA, USA). Transcripts were detected using cDNA as template by PCR with primers listed in DATASET_S3.

### Genotoxic sensitivity and mutator phenotype assays

Ultraviolet-B (UV-B) sensitivity assays were performed according to [48]. For the bleomycin sensitivity assay, ∼2500 protoplasts per well in a 24-well plate were chronically exposed to indicated doses of bleomycin on an agar regeneration medium. The protoplast regeneration rate was quantified after 72 h from three replicates in each dose. The frequency of spontaneous mutations at the *APT* locus in WT and *brca2Δ* mutants was analyzed as previously described [48].

### GT assay

The GT efficiency at the *APT* locus in the WT and *brca2Δ* mutants was measured as described previously [58]. *Physcomitrium patens* protoplasts were transformed with the following plasmids: pUbi-Cas9 (expressing Cas9) [59], psgRNA-PpAPT#2 (expressing guide RNA to target *APT* locus), and PpAPT-KO7 (donor matrix containing a G418 resistance cassette flanked by genomic *APT* sequences). The double resistance to 2-fluoroadenine (2FA) and G418 can be conferred by targeted integration of the PpAPT-KO7 cassettes at the *APT* locus or cassette integration elsewhere in the genome coupled with nonsense mutations at the *APT* locus. To estimate the HDR frequency versus non-HDR, transformants with the disrupted *APT* gene due to cassette insertion at the locus by HR or nonsense mutation via non-HDR pathways were first selected on PpNH4 supplemented with 10 μM 2FA, followed by another round of selection on media with 50 mg l^−1^ G418 (Duchefa Biochemie, The Netherlands). The *APT* locus-specific insertion was confirmed by PCR analysis with primers amplifying insertion borders or single-copy insertion [58]. The HDR frequency was calculated by estimating G418-resistant plants among 2FA-resistant transformants. Independently from HDR frequency measurements, the ratio of targeted integrations versus random integrations was calculated from the number of stable G418 resistant transformants obtained after three successive rounds of selection on 50 mg/l G418 followed by selection on 2FA to identify *APT* locus-specific insertion. G418-resistant plants showing amplification of cassette but not at the *APT* locus by PCR were considered random integrations (Supplementary Fig. S4A) [58].

### Cytological techniques

Meiotic chromosome spreads were prepared as previously described [48]. For RAD51 immunostaining, we adopted a method described for Arabidopsis root tip nuclei according to [60]. Briefly, 6-day-old *P. patens* protonema filaments from WT or *brca2Δ* were incubated with or without 5 μg/ml bleomycin for 3 h for DSB induction. *Physcomitrium patens* filaments were first fixed for 45 min in 4% paraformaldehyde in PME [50 mM piperazine-N,N’-bis(2ethanesulphonic acid) (PIPES), pH 6.9; 5 mM MgSO_4_; 1 mM ethylene glycol-bis(β-aminoethyl ether)-N,N,N′,N′-tetraacetic acid (EGTA)] followed by three washes in PME for 5 min. Then, filaments were digested for 30 min at 37°C in digestion buffer [1% (w/v) cellulase, 0.5% (w/v) cytohelicase, 1% (w/v) pectolyase in PME] and were washed three times for 5 min in PME. Digested cells were then squashed gently onto slides with a coverslip and immersed in liquid nitrogen. Slides were air-dried and stored at −80°C. RAD51 immunostaining with rabbit anti-PpRAD51 antibody [61] in 1:500 dilution along with 4',6-diamidino-2-phenylindole (DAPI) on the squashed cells with protocol used for Arabidopsis root tips [60]. Antirabbit secondary antibodies were conjugated with Alexa 488 (A27034; Thermo Fisher Scientific, Waltham, MA, USA) in 1:400 dilution in the Vectashield^®^ mounting medium. Images were obtained with a Zeiss AxioObserver microscope and were analyzed using Zen Blue software. Images of plants, spores, and regenerating protoplasts were acquired with a Zeiss AxioZoom.V16 using Zen Blue software for acquisition and analysis. Detailed nuclei images of RAD51 foci and live imaging of GFP-BRCA2 germinating spores were taken using a LEICA SP5 II AOBS Tandem HyD or a Leica TCS SP8 AOBS confocal laser scanning microscope.

### Coimmunoprecipitation and Y2H assays

The coimmunoprecipitation (CoIP) assays were performed on protein extract from the WT and GFP-BRCA2 protonema treated with or without 5 μg/ml bleomycin for 3 h and ground in liquid nitrogen to a fine powder. Total cell lysates were prepared by adding lysis buffer [50 mM Tris, pH 8.0, 150 mM NaCl, 0.1% Triton, 5% glycerol, 0.5% NP40, 0.5 mM ethylenediaminetetraacetic acid (EDTA), 1× protease inhibitor cocktail, 1× phosphatase inhibitor], with incubation on a rotating shaker for 30 min followed by centrifugation at 10 000 × *g* for 20 min at 4°C. For each CoIP, cell lysates corresponding to 5 mg total protein were incubated with GFP-Trap^®^ magnetic particles M-270 (ChromoTek GmbH & Proteintech, Planegg-Martinsried, Germany) for 2 h at 4°C. Protein–antibody complexes were recovered on magnetic particles after washing four times with lysis buffer. Protein complexes were then eluted with Laemmli buffer and migrated on sodium dodecyl sulphate–polyacrylamide gel electrophoresis. Proteins were detected by western blot analysis with rat anti-GFP monoclonal antibody (cat no. 3h9, ChromoTek GmbH & Proteintech, Planegg-Martinsried, Germany) or anti-PpRAD51 antibody [61].

AH109 and Y187 (Clontech, TakaRabio, Shiga, Japan) yeast haploid strains were transformed with constructs encoding Gal4BD and Gal4AD fusion proteins for Y2H assays. After mating the haploid yeast strains with opposite mating types on YPD plates, diploid yeast cells were selected on a dropout medium without leucine and tryptophan (SD/ -LW). Y2H protein interactions were detected by plating five-fold serial dilutions (0–10 000 fold) of each diploid on selective media depleted in leucine, tryptophan, histidine, and adenine (SD/ -LWH and SD/ -LWHA) for 3–5 days at 30°C. The strength of interaction was quantified by scoring each dilution that grew on the selective medium. Each dilution growing on SD/ -LWH or SD/ -LWHA was scored 0.05 or 0.1, respectively. The final score for each diploid was obtained by summing the individual scores, with 0.75 (0.25 + 0.5) being the maximum score for five dilutions growing on each selective medium without any self-activation.

### Protein expression and purification

The *P. patens* BRCA2 (PpBRCA2)-FL (amino acids 1–391), PpBRCA2-Nter (1–250), PpBRCA2-NterT138 (1–138), and PpBRCA2-Cter (251–391) proteins were expressed in *Escherichia coli* BL21-Rosetta (DE3) (CamR). Expression clones containing recombinant pET15b-PpBRCA2 (FL, Nter, and Cter) (AmpR) were grown at 37°C with chloramphenicol (25 μg/ml) and ampicillin (100 μg/ml) until the OD_600 nm_ reached 0.6. Recombinant protein expression was then induced by adding 0.2 mM Isopropyl β-D-1-thiogalactopyranoside (IPTG) for overnight at 20°C. After centrifugation (2200 × *g*, 10 min, 4°C), cells resuspended in lysis buffer [50 mM TrisHCl, pH 7.5, 300 mM NaCl, 5% glycerol, 40 mM imidazole, 1% Triton X-100, 1 mM Phenylmethylsulfonyl fluoride (PMSF), 1 mM Dithiothreitol (DTT)] were frozen in liquid nitrogen and lysed by sonication (Vibra-Cell™, VC 505) with proteases inhibitors (cOmplete™, EDTA-free Protease Inhibitor Cocktail, Roche). Insoluble material was removed by centrifugation at 18 500 × *g*, 30 min, and 4°C.

For PpBRCA2-FL, PpBRCA2-Nter and PpBRCA2-Cter purification, the clarified supernatant with 1 mM DTT was loaded onto a 5 ml HisTrap™ column (GE Healthcare) at a 2 ml/min flow rate using FPLC (Äkta pure, Cytiva). After washing with wash buffer (25 mM Tris–HCl, pH 7.5, 500 mM NaCl, 1 mM DTT, and 5% glycerol), proteins were eluted with an imidazole gradient (20 ml) with elution buffer (25 mM Tris–HCl, pH 7.5, 500 mM NaCl, 1 mM DTT, 5% glycerol, 500 mM imidazole). Fractions diluted to 50 mM NaCl were further purified via a 1 ml Resource^TM^ Q or Resource^TM^ columns (Cytiva) using a linear gradient of 0.1–1 M NaCl. Fractions containing the protein of interest were subjected to size exclusion chromatography on a Superdex^®^ 75 10/300 GL column (Cytiva) equilibrated with 25 mM HEPES, pH 7, 100 mM NaCl, 1 mM DTT (Supplementary Fig. S8). The PpBRCA2-NterT138 (aminoacids 1 to 138) was provided by the CIGEx platform (Supplementary Fig. S8).

The *P. patens* DMC1 (PpDMC1), and RAD51 (PpRAD51-2) proteins fused to an N-terminal His6-SUMO tag, were expressed in *E. coli* BL21-Rosetta (DE3)-pLysS (CamR) using *pCDF-His6-SUMO-DMC1* and *pCDF-His6-SUMO-RAD51-2*, constructs by adding 0.2 mM IPTG. Cells were lysed in the lysis buffer (100 mM Tris–HCl, pH 7.5, 3 M NaCl, 10% glycerol, 5 mM β-mercaptoethanol), and the clarified supernatant was loaded onto a 5 ml HisTrap™ column (GE Healthcare) using FPLC (Äkta pure, Cytiva). After washing with wash buffer (25 mM Tris–HCl, pH 8, 500 mM NaCl, 10% glycerol, 1 mM DTT, and 0.1 mM EDTA), proteins were eluted with an imidazole gradient (30 ml) in the wash buffer plus 400 mM imidazole. The SUMO-tag was cleaved by the SUMO protease [ratio of 200:1 (w:w)] overnight at 4°C during dialysis in 25 mM Tris–HCl, pH 8, 500 mM NaCl, 10% glycerol, 5 mM β-mercaptoethanol. The sample was reloaded on a HisTrap™ column to recover the tag-free proteins in the flow-through. The sample was further purified using a Hi-Trap™ heparin HP 5 ml column (GE Healthcare) with a 0.1–1 M NaCl linear gradient. Fractions containing the protein of interest were pooled and diluted to reach 100 mM salt concentration and reloaded on a 6 ml Resource™ Q column (Cytiva) equilibrated with 25 mM Tris–HCl, pH 8, 100 mM NaCl, 10% glycerol, 1 mM DTT. Fractions containing PpDMC1, PpRAD51-2, and PpBRCA2-NterT138 from elution with a 0.1–1 M NaCl linear gradient were pooled, aliquoted, and stored at −80°C (Supplementary Fig. S8). Thermal shift analysis, electron microscopy, and electrophoretic mobility shift assays (EMSAs) were used to determine if the purified PpDMC1 was active (Supplementary Fig. S13).

### Nuclear magnetic resonance

For nuclear magnetic resonance (NMR) analysis, ^15^N-labeled proteins were produced in BL21-Rosetta (DE3) (CamR) grown in M9 medium containing 0.5 g/l ^15^NH_4_Cl and 2 g/l ^12^C-glucose. 2D NMR ^1^H-^15^N SOFAST-HMQC experiments were conducted on a 3 mm sample tube containing ^15^N-labeled PpBRCA2, PpBRCA2-Nter, and PpBRCA2-Cter at 20, 200, and 260 μM concentrations, respectively, in 25 mM HEPES, pH 7.0, 100 mM NaCl, with or without 1 mM DTT, using a 950-MHz Bruker Advance III spectrometer with a triple resonance cryogenic TCI probe at 280 K.

### BioLayer interferometry (BLI)

BioLayer Interferometry (BLI) between recombinant recombinases and biotinylated peptides, as well as DNA substrates, were analyzed at 25°C using BLI using an Octet RED96 instrument (ForteBio, USA). Biotinylated PpBRCA2 peptide sequences are given in DATASET_S3 and were synthesized by ProteoGenix, France.

Briefly, the biosensors were first hydrated for 10 min in buffer A (25 mM Tris, pH 7.5, 100 mM Na_2_SO_4_, 5 mM β-mercaptoethanol) for PpRAD51-2 and PpDMC1 or buffer B (25 mM Tris, pH 7.5, 100 mM NaCl, 5 mM β-mercaptoethanol) for PpDMC1 only. The 0.5 μM biotinylated peptides solution was used for immobilization on Streptavidin (SA) biosensors. To monitor the association, prewashed biosensors were incubated with PpRAD51-2 for 300 s in buffer A supplemented up to 0.05% Tween-20. Kinetic assays were performed at varying concentrations of PpRAD51-2 with two-fold serial dilutions (except for the peptide BRC3G). For PpDMC1, SA biosensors were incubated with 10 μM protein for 300 s. The dissociation was followed for 600 s, and the dissociation constants (Kd) were determined in steady-state mode. After each association and dissociation round, biosensors underwent five regeneration cycles for 10 sec in 1 M NaCl or 5 M NaCl for BRC3G peptide, followed by 10 sec in buffer A or B with 0.05% Tween-20.

For the interactions between PpBRCA2-FL, PpBRCA2-Nter, PpBRCA2-NterT138, PpBRCA2-Cter, and biotinylated ssDNA [100 nucleotide (nt)], the SA biosensors were hydrated in 25 mM Tris, pH 7.5, 100 mM NaCl or 25 mM HEPES, pH 7, 100 mM NaCl, 1 mM DTT buffer for 10 min. The 50 nM biotinylated DNA (100 nt ssDNA) solution was used for immobilization on SA biosensors. The binding buffers were supplemented with up to 0.1% Tween-20. After washing, sensors were incubated with PpBRCA2-FL, PpBRCA2-Nter, PpBRCA2-NterT138, and PpBRCA2-Cter at varying concentrations with two-fold serial dilutions for 300 s followed by 600 s to monitor association and dissociation, respectively. Biomolecular interactions were measured between PpRAD51-2 and 6His-PpBRCA2-Cter immobilized on Ni-NTA biosensors, prehydrated in 25 mM Tris, pH 7.5, 100 mM NaCl, 1 mM DTT buffer for 10 min. The binding assays were performed in buffer A supplemented with 0.1% Tween-20. After washing, sensors were incubated with PpRAD51-2 at varying concentrations for 300 s followed by 600 s to monitor association and dissociation cycle.

### Electrophoretic mobility shift assay

The PpDMC1-ssDNA filament assembly was performed using 1 μM PpDMC1 with 3 μM Cy5-labeled 100 nt ssDNA substrate for 15 min at 37°C in buffer C (10 mM Tris–HCl, pH 7.5, 50 mM NaCl, 2 mM MgCl_2_, 2 mM CaCl_2_, 2 mM ATP, and 1 mM DTT). The PpRAD51-2-ssDNA filament assembly was performed using 0–4 μM of either PpBRCA2-Cter or BRC4 with 3 μM ssDNA (100 nt) Cy5-labeled DNA substrate for 15 min at 37°C in buffer D (10 mM Tris–HCl, pH 7.5, 50 mM NaCl, 5 mM MgCl_2_, 2 mM ATP, and 1 mM DTT). The PpBRCA2-DNA binding assays were conducted using 2 μM Cy5-labeled of either ssDNA (100 nt), dsDNA (250 bp), or a ss–dsDNA overhang (150 nt–100 bp) substrates with 0–2.4 μM of PpBRCA2-FL, PpBRCA2-Cter, PpBRCA2-Nter, or PpBRCA2-NterT138 for 7 min at 37°C in buffer D. For all reactions, protein–DNA complexes were fixed by adding 0.01% glutaraldehyde for 5 min at room temperature. The reaction products were run onto a 0.75% agarose gel in 0.5× Tris-acetate-EDTA at 80 V and 4°C for 20 min. The gel was scanned on a Typhoon imager (GE Healthcare Life Science) or a ChemiDoc imager (Bio-Rad) using the Cy5 channel. The percentage of DNA–protein complexes was estimated in each condition by ImageJ software, measuring the intensity of the displaced bands (corresponding to the DNA–protein complexes) normalized to the total intensity of all bands (corresponding to free DNA plus DNA–protein complexes).

The thermal stability measurement by fluorescence was assessed with 20 μM of PpDMC1 in 25 mM Tris–HCl, pH 7.5, 300 mM NaCl, 5 mM ß-mercapthoethanol loaded on a 10 μl capillary, using a Tycho instrument (NanoTemper, Germany).

### Negative staining electron microscopy

The PpDMC1 protein self-assembly and PpDMC1-ssDNA filament architecture were observed using negative staining in bright-field mode for electron microscopy. For this, 5 μl of PpDMC1 protein at 0.5 mg/ml in 25 mM Tris, pH 8.0, 600 mM NaCl, 10% glycerol, and 1 mM DTT was plated on a grid. PpDMC1-ssDNA filaments were assembled using 1 μM DMC1 with or without 3 μM ssDNA (100 nt long) in buffer C at 37°C for 15 min. A drop of the PpDMC1 protein or filament reaction was directly deposited on a carbon-coated copper grid pre-activated with glow discharge (plasma). Grids were rinsed and negatively stained with aqueous 2% (w/v) uranyl acetate, dried carefully with a filter paper. Grids were observed in canonical bright-field imaging by Tecnai 12 Spirit (Thermo Fisher) transmission electron microscope with a K2 camera (Gatan).

### AlphaFold3 predictions

AlphaFold3 predictions for protein complexes were computed at https://alphafoldserver.com/welcome, which provided plDDT and ipTM scores and graphs. Molecular graphics and analyses were performed with the UCSF ChimeraX (version 1.7.3), developed by the Resource for Biocomputing, Visualization, and Informatics at the University of California, San Francisco.

## Results

### Identification of a noncanonical BRCA2 homolog in *P. patens*

**Figure 1.**
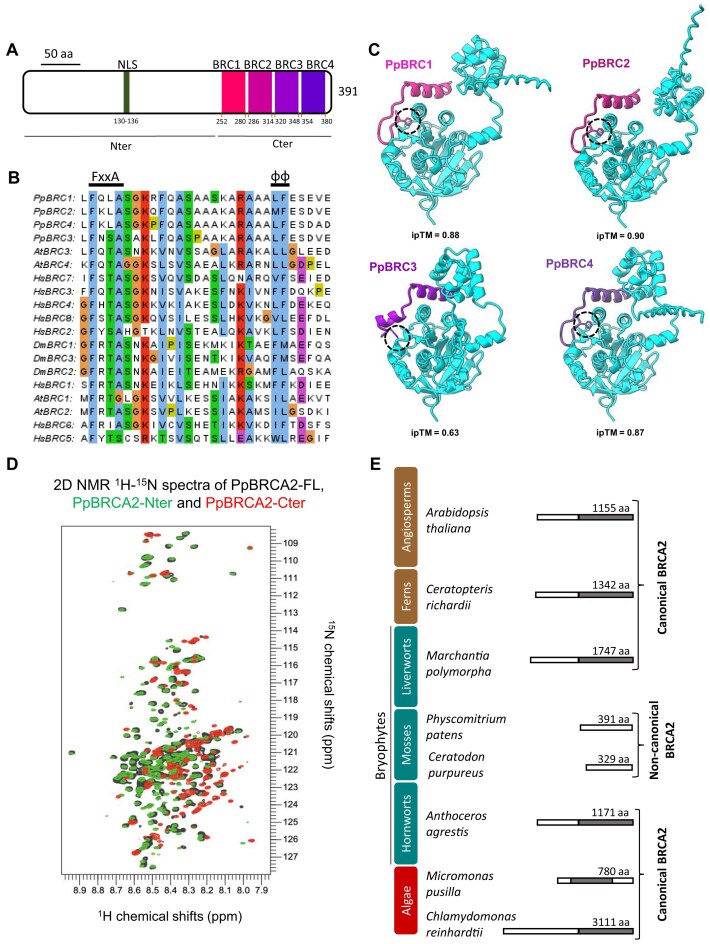
Identification of noncanonical BRCA2 homolog in *P. patens*. (**A**) Schematic representation of *P. patens* BRCA2 (PpBRCA2) with predicted NLS in green and four conserved BRC repeats in pink, orchid, violet and purple. (**B**) Alignment of BRC repeats from *Homo sapiens (Hs), Arabidopsis thaliana (At), D. melanogaster (Dm)*, and *P. patens (Pp)*. Conserved FxxA and dihydrophobic (φφ) motifs are labeled with amino acid residues in Clustal X color code. (**C**) 3D structure models of the interaction between PpRAD51-1 (cyan) and PpBRCA2 BRC1(pink), BRC2 (orchid), BRC3 (violet), BRC4 (purple) built with AlphaFold3. The phenylalanine of the conserved FxxA motif in BRC repeats is shown in dotted circles. The ipTM scores assessing the accuracy of the predicted interfaces are presented under each model. (**D**) Superimposition of the 2D NMR ^1^H ^15^N SO-FAST HMQC spectra of PpBRCA2 (black), PpBRCA2-Nter (green), and PpBRCA2-Cter (red) recorded at 950 MHz, showing that all constructs are intrinsically disordered. (**E**) Evolutionary conservation of canonical BRCA2 homologs with DBD in gray or noncanonical BRCA2 without DBD in algae (red), bryophytes (cyan), and tracheophytes (brown) of the Plantae kingdom.

BRCA2 orthologs are present in diverse plants but were not identified in *P. patens* [41, 29], a moss model species from the bryophyte clade in the Plantae kingdom. Among plants, two *A. thaliana* AtBRCA2A and AtBRCA2B homologs possess three key features of eukaryotic BRCA2 orthologs: BRC repeats, a canonical DBD, and a putative nuclear localization signal (NLS) [7, 29, 62]. We performed PSI-BLAST searches with AtBRCA2A as a query but failed to identify any homologs in *P. patens*. We employed a more sensitive sequence search method, HHpred, to detect remote homology based on the comparison of profile hidden Markov models [52]. Using AtBRCA2A as bait and targeting HHpred searches to human, drosophila, and moss proteomes with the default PDB_mmCIF70_16_Aug database, we retrieved 147 hits including human and drosophila BRCA2 homologs as well as other DBD-containing proteins such as RPA (DATASET_S1). Among the *P. patens* hits, proteins without BRC repeats were ruled out to be BRCA2 homologs. We detected only one sequence corresponding to the protein XP_001764308.1/Pp6C10_10830 with a probability of 94.7% and a pairwise sequence identity of 31% with the N-terminal region of AtBRCA2A (first 506 amino acids). The HHpred pairwise alignment revealed the presence of BRC repeats in Pp6C10_10830 (Supplementary Fig. S1A). Conversely, HHpred search using Pp6C10_10830 retrieved BRCA2 homologs from Arabidopsis, human, and drosophila with pairwise alignments showing strong homology in the BRC repeat region, substantiating that Pp6C10_10830 is homologous to other BRCA2 proteins (DATASET_S2). The *P. patens Pp6C10_10830* gene consists of a single exon without any intron located on chromosome 10 and encodes a 391 amino acids (aa) long protein, which we designated as PpBRCA2 (Supplementary Fig. S1B). PpBRCA2 possesses a NLS in its N-terminal region, and four BRC repeats packed one after the other in its C-terminal region (Fig. 1A and Supplementary Fig. S1A and B). The alignment of BRC repeats detected fully conserved FxxA and dihydrophobic motifs across the moss, *A. thaliana*, human, and drosophila (Fig. 1B), corresponding to previously reported two modules of BRC repeats [32, 63]. Alphafold3 (AF) [64] predicted interactions with strong confidence scores (ipTM > 0.7) between moss BRC repeats 1, 2, and 4 and both moss RAD51 homologs (Fig. 1C and Supplementary Fig. S1C). The superimposition of calculated models and the crystal structure available for human BRC4 bound to RAD51 revealed that the conserved phenylalanine residue of the FxxA motif is buried within the RAD51 ATPase domain (Supplementary Fig. S1D) [65]. The BRC3–RAD51 interaction scores were lower (ipTM < 0.7), with the phenylalanine of the FxxA motif not positioned in the ATPase domain of RAD51 (Fig. 1C and Supplementary Fig. S1C). These AF models are consistent with previous reports showing differences in interaction affinities of BRC repeats towards RAD51 [66, 67, 30].

Strikingly, the HHpred pairwise alignment revealed that PpBRCA2 lacks a canonical DBD, which typically includes a well-folded helical domain and three OB domains (Fig. 1A, and Supplementary Fig. S1E and F). We purified recombinant ^15^N-labeled full-length PpBRCA2-FL, PpBRCA2-Nter (aa 1–250 aa), and PpBRCA2-Cter (251–391 aa) to assess the presence of folded domains by NMR spectroscopy. We recorded 2D NMR ^1^H ^15^N fingerprints, in which each peak represents one residue, and the position of the peak in the spectrum reflects its chemical environment. We did not assign the NMR peaks to the BRCA2 residues. However, we observed that the fingerprint of PpBRCA2-FL corresponds to the superimposition of the fingerprints of PpBRCA2-Nter and PpBRCA2-Cter, showing that these two regions have independent conformations. Also, all peaks were detected within a narrow ^1^H chemical shift range, indicating that recombinant PpBRCA2-FL, PpBRCA2-Nter and PpBRCA2-Cter are highly disordered in solution (Fig. 1D). The repeated BRC sequences gave rise to stretches of partially overlapping peaks, suggesting that they have similar disordered conformations (Fig. 1D). Altogether, our NMR analysis demonstrated the absence of a well-folded DBD in PpBRCA2.

Among the well-annotated moss genomes, PpBRCA2 homologs without DBD were identified using PSI-BLAST searches in *Ceratodon purpureus* (CepurR40.9G141900 or CepurGG1.9G149800) and *Funaria hygrometrica* (Fh_23015) [68] species, indicating that loss of DBD is common to at least three moss genera (Fig. 1E and Supplementary Fig. S1E). Since establishing phylogenetic relationships among BRCA2 orthologs is challenging due to varying protein sizes and low aa conservation outside the BRC repeats and DBD [34], we compared the presence or absence of the canonical DBD across various plant clades. BRCA2 homologs with canonical DBD were readily detected in the clades of green algae, ferns, and angiosperms, as well as in both liverworts and hornworts – which together with mosses form the bryophyte group, suggesting that BRCA2 homologs without a canonical DBD have likely emerged in moss lineages only (Fig. 1E). Overall, two variants of BRCA2 orthologs appear to exist: canonical BRCA2 with a canonical DBD and noncanonical BRCA2 homologs lacking this DBD (Supplementary Fig. S1F).

### PpBRCA2 is essential for normal plant growth and programmed DSB repair during meiosis

**Figure 2.**
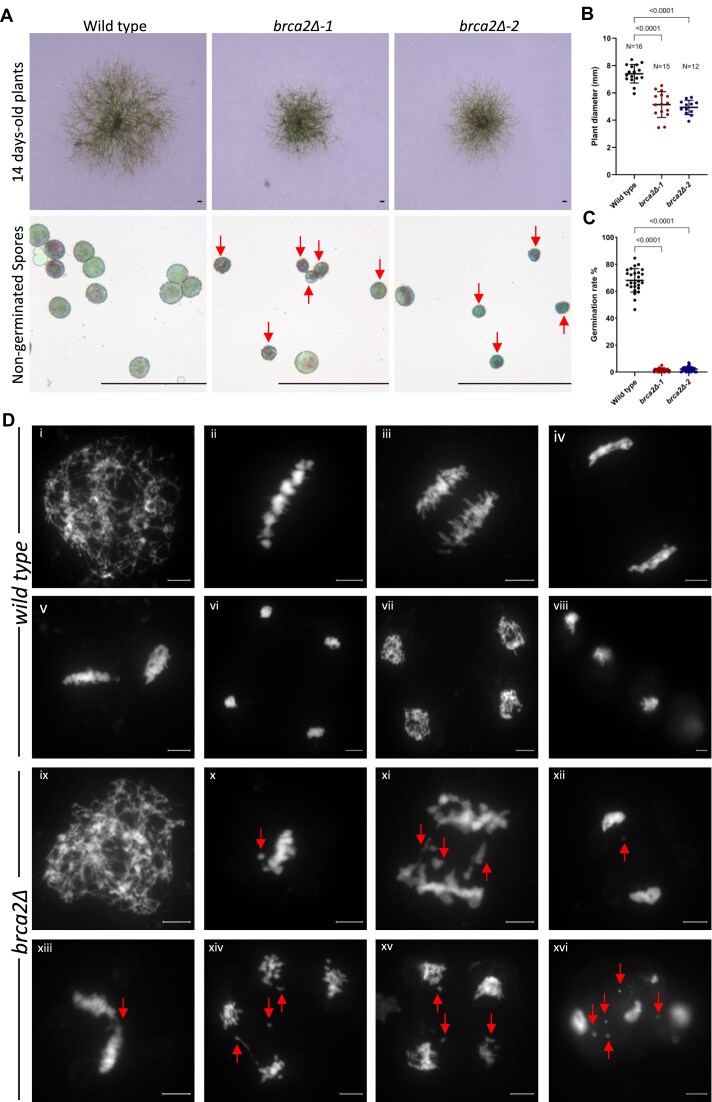
*Physcomitrium patens brca2Δ* mutants display growth retardation, nonviable spores, and meiosis defects. (**A**) Top row: Representative images of 14-day-old WT and *brca2Δ* plants on minimal media. Scale bars: 1 mm. Bottom row: Images of nongerminated WT and *brca2Δ* spores stained with Alexander stain. Red arrows point to an anomaly in the shape and size of mutant spores. Scale bars: 50 μm. (**B**) Quantification of 14-day-old plant diameter on minimal media. Each dot represents the diameter of an individual plant for WT, *brca2Δ-1*, and *brca2Δ-2* genotypes. Horizontal bars indicate the mean and standard deviation. The *P*-values were calculated using one-way analysis of variance (ANOVA) with Dunnett’s test for multicomparison. (**C**) Quantification of spore germination rate after 6 days of growth. Spores were pooled from four different capsules for each genotype. Each dot represents the germination percentage of spots containing 8–25 spores, with total spore counted in WT = 3357, *brca2Δ-1*= 2093, and *brca2Δ-2*= 1715. Horizontal bars indicate the mean and standard deviation. The *P*-values obtained using one-way ANOVA with Dunnett’s test for multicomparison. (**D**) Representative images of DAPI-stained chromosome spreads at leptotene (i, ix), metaphase I (ii, x), anaphase I (iii, xi), telophase I (iv, xii), metaphase II (v, xiii), anaphase II (xiv), telophase II (vi, vii, xiv), and mature spores (viii, xvi) in WT (i–viii) and *brca2Δ* (ix–xvi). Red arrows indicate lagging chromosomes, DNA fragmentation, or univalent chromosomes. Scale bars: 5 μm.

The life cycle of *P. patens* involves haploid spores developing into filamentous protonema, leading to gametophores on leafy shoots, which produce diploid sporophytes upon fertilization. Meiosis occurs in the sporophyte and generates haploid spores to complete the life cycle. We explored the function of PpBRCA2 by analyzing four deletion mutants generated using CRISPR-Cas9 genome editing in Reute (*brca2Δ-1* and *brca2Δ-2*) and Gransden (*brca2Δ-3* and *brca2Δ-4*) *P. patens* ecotypes (Supplementary Fig. S2A). All four *brca2Δ* mutants were viable but exhibited a significant reduction (∼30%) in plant diameter compared with WT (Dunnett’s test P <.0001), indicating a slower protonema growth in the absence of PpBRCA2 (Fig. 2A and B and Supplementary Fig. S2D). The reduced growth was also observed in *rad51-1rad51-2* mutants (Supplementary Fig. S2A–B). Both WT and *brca2Δ* mutants developed sporophyte and produced spores; however, *brca2Δ* mutant spores were irregularly shaped, heterogeneous in size, and generally smaller than the round, larger WT spores (Fig. 2A and Supplementary Fig. S2C). Spore germination test showed that only 2% of *brca2Δ* spores germinated compared with 70% of WT spores, suggesting that *brca2Δ* mutants predominantly produce nonviable spores (Fig. 2C and Supplementary Fig. S2E–G). The few germinated *brca2Δ* spores displayed abnormal protonema with severely reduced growth, potentially due to aneuploidy (Supplementary Fig. S2B). Thus, PpBRCA2 is essential for normal development and spore viability.

*Physcomitrium patens* haploid spores are the direct product of meiosis in the sporophytes, suggesting that nonviable *brca2Δ* spores may result from defective meiosis [48]. We next analyzed the meiotic progression in WT, *brca2Δ-1*, and *brca2Δ-2* sporophytes by preparing meiotic chromosome spreads stained with DAPI. In WT, meiotic chromosomes undergo DSB formation and HDR-mediated repair during prophase I, culminating in bivalent formation at metaphase I, where homologous chromosomes are held together by COs (Fig. 2D). These bivalents ensure the balanced segregation of homologous chromosomes at anaphase I (Fig. 2D). During the second meiotic division, sister chromatids align at metaphase II and separate at anaphase II (Fig. 2D). Following telophase II and cytokinesis, four haploid cells (tetrad) form, completing meiosis and leading to spore production (Fig. 2D). We observed that ∼95% of metaphase I cells (*N* = 43) displayed only bivalents, while <5% of cells (*N* = 2) showed a mixture of bivalents and univalents (chromosomes without CO) (Supplementary Fig. S3D and Supplementary DATASET_S3). In *brca2Δ* mutants, we detected a significantly high frequency of aberrant metaphase I cells (∼21%, *N* = 12 out of 56), exhibiting DAPI-stained chromatin that remained outside the aligned bivalents (Fig. 2D, Supplementary Fig. S3D, and Supplementary DATASET_S3). Due to high chromatin compaction and low resolution, we were unable to determine whether these signals represented univalents or fragmented chromosomes. However, we consistently observed chromosome bridges and micronuclei in later meiotic stages and mature spores (N = 81) (Fig. 2D, Supplementary Fig. S3D, and Supplementary DATASET_S3), indicating severe meiotic defects in *brca2Δ*. These abnormalities suggest a failure to repair meiotic breaks, leading to unbalanced chromosome segregation in *brca2Δ* mutants. Similar defects were also observed in *rad51-1rad51-2* mutant meiocytes (Supplementary Fig. S3C-D and Supplementary DATASET_S3). Taken together, PpBRCA2 is required for DSB repair during meiosis in *P. patens*.

### PpBRCA2 is essential for somatic DSB repair and genome stability

**Figure 3.**
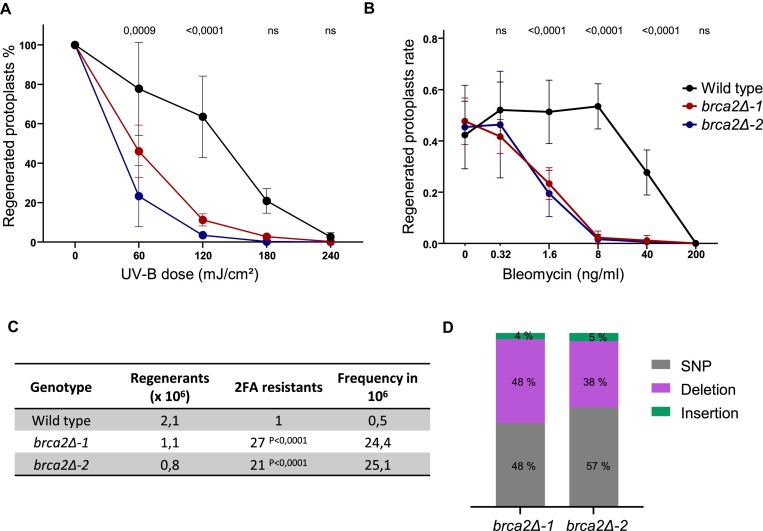
Hypersensitivity to genotoxic stress and spontaneous mutation rate in WT and *brca2Δ*. (**A**) Survival curves of regenerated WT (black), *brca2Δ-1* (red), and *brca2Δ-2* (blue) protoplasts 6 days post-exposure to UV-B. Values are normalized on the nontreated sample. For each point, the mean of three independent experiments is plotted with error bars indicating standard deviation. The *P*-values were computed using two-way ANOVA with Dunnett’s test for multicomparison. (**B**) Survival curves of regenerated WT (black), *brca2Δ-1* (red), and *brca2Δ-2* (blue) protoplasts after 3 days of constant exposure to bleomycin. For each point, the mean of three independent experiments is plotted with error bars indicating standard deviation. The *P*-values were obtained using two-way ANOVA with Dunnett’s test for multicomparison. (**C**) The frequency of spontaneous mutations at the *APT* locus in regenerated WT and *brca2Δ* protoplasts. The *P*-values were calculated using the Fisher’s exact test. (**D**) Histograms show the distribution of single nucleotide polymorphism (SNP), deletions, or insertions identified by sequencing in *brca2Δ-1* and *brca2Δ-2* at the *APT* locus.

The role of PpBRCA2 in somatic DSB repair was determined by analyzing the sensitivity of *brca2Δ* mutants to different DNA-damaging agents. We exposed WT and *brca2Δ* (*brca2Δ-1, -2, -3*, and *-4*) regenerating protoplasts to various dosages of UV-B that result in pyrimidine dimers, leading to single- and double-strand breaks during replication [69]. The *brca2Δ* mutants showed increased sensitivity to UV-B, as evidenced by significantly lower survival rate compared with WT at 60 and 120 mJ/cm² (Fig. 3A and Supplementary Fig. S4A, C, and E). We next examined sensitivity to chronic exposure to bleomycin that induces DSBs [70] by plating WT and *brca2Δ* protoplasts on media supplemented with increasing concentrations of bleomycin (0.32–200 ng/ml) and monitoring cell regeneration for 72 h. A severe reduction in the frequency of regenerated protoplasts in *brca2Δ* mutants at 1.6, 8, and 40 ng/ml indicated hypersensitivity to chronic DSBs and supported a role of PpBRCA2 in DSB repair (Fig. 3B and Supplementary Fig. S4B, D, and F). These findings demonstrate that PpBRCA2 is essential for DSB repair in *P. patens*.

We next evaluated genetic instability upon the loss of PpBRCA2 by measuring the spontaneous mutation frequency at the *APT* (adenine phosphoribosyl transferase) reporter gene as previously described [71]. The 2FA is a toxic adenine analog for WT but allows the selection of plants harboring nonsense mutations in the *APT* gene. A total of 2.1 × 10^6^, 1.1 × 10^6^, and 0.8 × 10^6^ protoplasts plated on media containing 10 μM 2FA resulted in 1, 27, and 21 2FA-resistant plants in WT, *brca2Δ-1*, and *brca2Δ-2*, respectively. This corresponded to a spontaneous mutation frequency of 0.5 × 10^6^ in WT, 24.4 × 10^6^ in *brca2Δ-1*, and 25.1 × 10^6^ in *brca2Δ-*2, indicating a significant ∼50-fold increase in mutation accumulation frequency at *APT* locus in *brca2Δ* mutants compared with WT (Fig. 3C). Sequencing analysis in 2FA-resistant *brca2Δ* plants revealed that half of the mutations were SNPs, leading to amino acid changes with premature stop codons, or splicing site alterations (Fig. 3D and Supplementary DATASET_S3). Other mutations were deletions, ranging from 1 to 283 bp, while <5% of resistant plants showed insertions, mostly 1–2 bp, except one being a 37-bp insertion (Fig. 3D and Supplementary DATASET_S3). Notably, all insertions except one were accompanied by deletions, often displaying 2–4 bp microhomology at the repair junctions. The only WT-resistant plant carried an SNP affecting a splice site at the exon 3 border (Fig. 3D and Supplementary DATASET_S3). These findings support a crucial role of PpBRCA2 in repairing naturally occurring DNA damage and in preventing genome-wide mutation accumulation, a conserved function of BRCA2 orthologs [5].

### Loss of PpBRCA2 disrupts somatic HDR and GT

**Figure 4.**
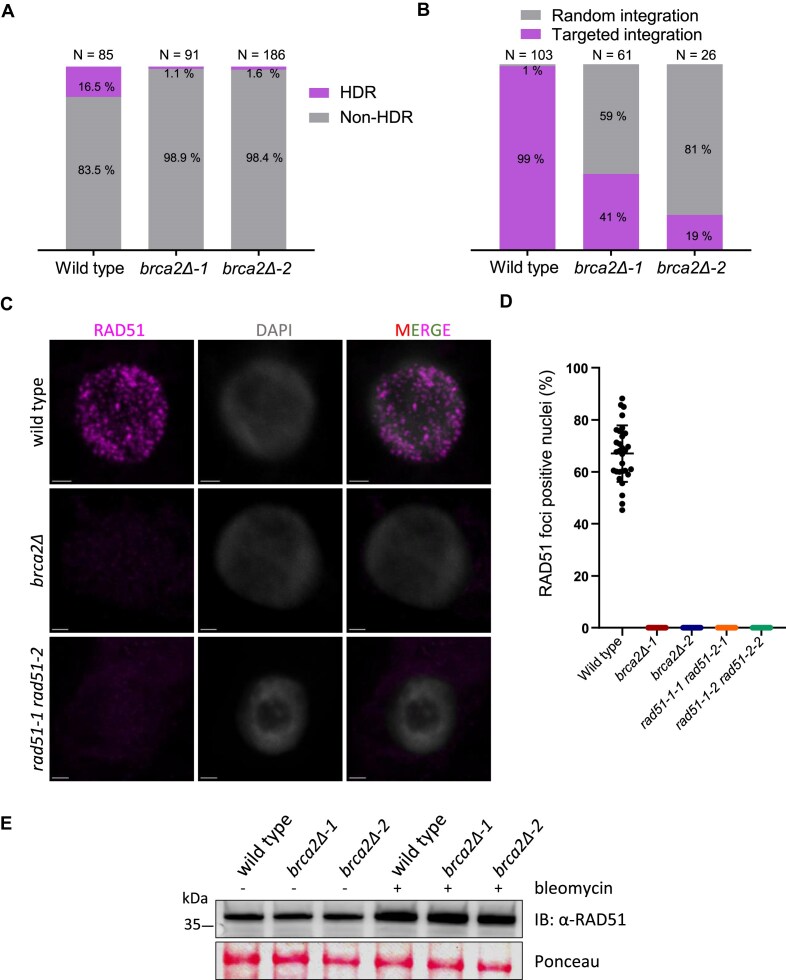
Analysis of homologous recombination repair (HDR) and RAD51 recruitment in *P. patens* WT and *brca2Δ* mutants. (**A**) Percentage of GT events (donor G418 cassette insertion) by HDR (purple) and non-HDR events (no insertion) in gray at the *APT* locus in WT, *brca2Δ-1*, and *brca2Δ-2* plants. Cassette integrations were confirmed by genotyping G418^R^ and 2FA^R^ plants using *APT*-locus-specific primers. (**B**) Percentage of targeted (purple) and random integration (gray) of the donor G418 cassette at the *APT* locus in WT and *brca2Δ* plants. Cassette integrations were confirmed by genotyping stable G418^R^ plants using *APT*-locus-specific primers. (**C**) Immunolocalization of RAD51 (purple) and DNA stained with DAPI (gray) on squashed protonema cells from WT, *brca2Δ*, and *rad51-1-2* plants treated with 5 μg/ml bleomycin for 3 h. Scale bars: 2 μm. (**D**) Percentage of RAD51 foci positive cells from WT, *brca2Δ*, and *rad51-1-2* plants. Each dot represents the percentage of RAD51-positive nuclei from various images, with total cells counted in WT = 1698, *brca2Δ-1*= 1083, *brca2Δ-2*= 1554, *rad51-1 rad51-2-1*= 385, and *rad51-1 rad51-2-2*= 404. Horizontal bars indicate the mean and standard deviation. (**E**) Western blot analysis of RAD51 steady-state levels from WT and *brca2Δ* moss protonema total protein extract with or without 3 h treatment with 5-μg/ml bleomycin probed with anti-RAD51 antibody. Ponceau staining is used as loading controls for each well.

Given the conserved role of BRCA2 orthologs in HDR, we assessed homology-dependent GT efficiency at the *APT* locus in WT and *P. patens brca2* mutants. We co-transfected protoplasts with three plasmids: one expressing the Cas9 gene, the second expressing the sgRNA#2 targeting the *APT* gene, and the third carrying a donor DNA cassette with a G418-resistant gene flanked by the *APT* gene sequences homology arms (Supplementary Fig. S5) [58]. The DSB repair at the *APT* locus in our GT assay can generate three genotypes: [1] 2FA-resistant and G418-sensitive plants can arise from non-HDR [e.g. classical NHEJ or alternative end-joining (Alt-EJ)] due to mutations in the *APT* gene with no cassette integration. [2] 2FA- and G418-resistant plants can originate from HDR-mediated GT with the donor G418-cassette integration at the *APT* locus. [3] 2FA- and G418-resistant plants can derive from mutations in the *APT* gene by non-HDR and with illegitimate donor cassette integration elsewhere in the genome. Following the protoplast transfection, plants were selected on 2FA and tested for G418 resistance. The majority of plants (83.5% in WT and 98.4% in *brca2Δ* mutants) were 2FA-resistant with no G418 cassette integration, likely resulting from NHEJ or Alt-EJ repair (Fig. 4A), suggesting *PpBRCA2* is not essential for non-HDR pathways. However, the GT rate—measured as the frequency of 2FA- and G418-resistant plants—was ∼10-fold lower in *brca2Δ* mutants (1.6%) than in WT (16.5%), similar to *rad51-1 rad51-2* [58]. This demonstrates that PpBRCA2 is essential for HDR-mediated GT.

Since HDR impairment enhances random DNA integration in *P. patens* [48, 58], we also examined the frequency of targeted versus random integration of the donor cassette by selecting co-transfected WT and *brca2Δ* protoplasts on G418 first and then testing for 2FA resistance. Targeted integration was confirmed by the PCR-detected cassette insertion at the *APT* locus, while random integrations lacked the *APT* locus-specific integration in stable G418-resistant plants (Supplementary Fig. S5). In WT, 99% of G418-resistant plants had targeted integration events, with only 1% showing random integration. In contrast, *brca2Δ* plants exhibited a severe reduction in targeted integration events (<41%), indicating a higher rate of random insertions (Fig. 4B). Our data argue that PpBRCA2 promotes HDR to counteract illegitimate integration of exogenous DNA and reinforce the importance of PpBRCA2 for genome stability in *P. patens*.

### PpBRCA2 is essential to RAD51 focus formation

After observing defective HDR in *brca2Δ* mutants, we examined whether PpBRCA2 mediates the recruitment of RAD51, detectable as nuclear foci following DSB induction. Protonema filaments treated with or without 5 μg/ml of bleomycin for 3 h were immunostained with anti-RAD51 antibody and DAPI. Untreated WT, *brca2Δ*, and *rad51-1 rad51-2* cells showed no RAD51 foci (Supplementary Fig. S3E). No signal in bleomycin-treated *rad51-1 rad51-2* indicated that our anti-RAD51 antibody is specific to RAD51 (Fig. 4C). Numerous RAD51 nuclear foci were observed in ∼70% of bleomycin-treated WT protonema cells (Fig. 4C and D). In contrast, the bleomycin-treated *brca2Δ* protonema cells showed no RAD51 focus formation (Fig. 4C and D). The lack of RAD51 foci could be attributed to low protein levels or a recruitment defect in *brca2Δ*. However, our western blot analysis revealed similar RAD51 protein abundance in bleomycin-treated WT, *brca2Δ-1*, and *brca2Δ-2* plants (Fig. 4E), suggesting that despite producing WT levels of RAD51, *brca2Δ* mutants fail to recruit RAD51 at DSB sites. These results demonstrate that, similar to other BRCA2 orthologs, PpBRCA2 is essential for RAD51 focus formation in response to DSBs.

### A canonical *A. thaliana* BRCA2 cannot complement *P. patens brca2Δ*

**Figure 5.**
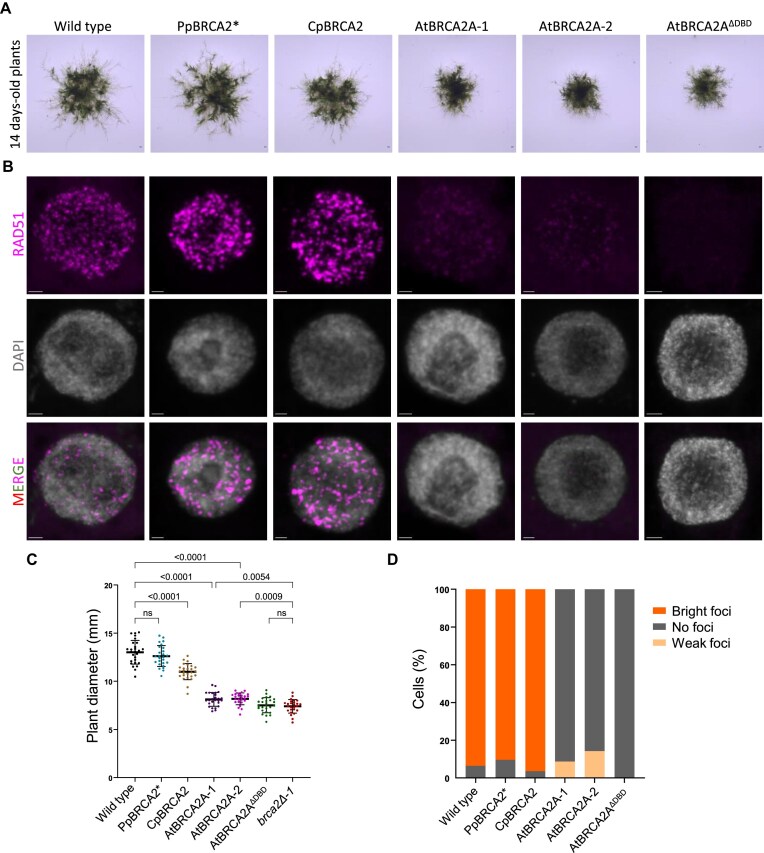
Growth phenotype and RAD51 recruitment in *P. patens brca2Δ-1* complemented plants. (**A**) Representative images of 14-day-old WT, PpBRCA2* (*brca2Δ-1*), CpBRCA2 (*brca2Δ-1*), and AtBRCA2^ΔDBD^ (*brca2Δ-1*) plants. Scale bars: 1 mm. (**B**) Quantification of 14-day-old plant sizes on minimal media. Each dot represents the diameter of an individual plant for WT, PpBRCA2*, *CpBRCA2*, *AtBRCA2A-1*, *AtBRCA2A-2*, and *AtBRCA2^ΔDBD^* genotypes. Horizontal bars indicate the mean and standard deviation. The *P*-values were calculated using one-way ANOVA with Dunnett’s test for multicomparison, ns; nonsignificant. (**C**) Immunolocalization of RAD51 (purple) and DAPI staining (gray) on the squashed protonema cells of WT, PpBRCA2*, *CpBRCA2*, *AtBRCA2A-1*, *AtBRCA2A-2*, and *AtBRCA2^ΔDBD^* plants treated with 5 μg/ml bleomycin for 3 h. Scale bars: 2 μm. (**D**) Quantification of RAD51 foci on squashed protonema cells of WT and BRCA2-complemented plants. The percentage of RAD51 foci-positive cells is shown for each genotype. Bright foci indicate signal as seen in WT, PpBRCA2*, and *CpBRCA2*, while weak foci indicate signal as seen in *AtBRCA2A-1* and *AtBRCA2A-2*. The total cells were analyzed in: WT = 1352, PpBRCA2* = 755, *CpBRCA2*= 839, *AtBRCA2A-1* = 729, *AtBRCA2A-2* = 690, and *AtBRCA2^ΔDBD^*= 1449. Horizontal bars indicate the mean and standard deviation. The *P*-values were calculated using one-way ANOVA with Dunnett’s test for multicomparison, ns; nonsignificant.

*Arabidopsis thaliana* BRCA2 (*AtBRCA2*) is a well-characterized canonical homolog from the angiosperm lineage [7]. Despite *A. thaliana* and *P. patens* diverging nearly 450 million years ago, both species retain an evolutionarily conserved machinery to repair DNA damage via HDR, including RAD51 and DMC1 recombinases. We tested whether a canonical BRCA2 could functionally replace a noncanonical BRCA2 by integrating full-length *AtBRCA2A*, truncated *AtBRCA2A^ΔDBD^* without DBD, or noncanonical *C. purpureus BRCA2* (*CpBRCA2*) at the *PpBRCA2* locus in *brca2Δ-1*. As a control, we integrated the *PpBRCA2* gene with 15 silent polymorphisms (PpBRCA2*), to distinguish it from the WT *PpBRCA2* (Supplementary Fig. S6A). We isolated independent lines with the integration of each *BRCA2* gene at the *PpBRCA2* locus by genotyping and sequencing, and evaluated each line for restoration of the growth phenotype of *brca2Δ-1*. PpBRCA2* restored the WT-level growth in *brca2Δ-1* (Fig. 5A and C). The *CpBRCA2* integrated plants showed about 10% reduction in diameter compared with WT (Fig. 5A and C), largely rescuing the *brca2Δ-1* growth phenotype. However, neither *AtBRCA2A* nor *AtBRCA2A^ΔDBD^*integration rescued the *brca2Δ-1* growth defect to the WT-level, suggesting full-length or truncated *AtBRCA2A* failed to complement *brca2Δ-1* (Fig. 5A and C). We next assessed the RAD51 recruitment functionality of PpBRCA2*, CpBRCA2, *AtBRCA2A*, and *AtBRCA2A^ΔDBD^*using immunolocalization in protonema cells treated with 5μg/ml of bleomycin for 3 h. RAD51 foci were detected in >90% of cells in WT, PpBRCA2*, and *CpBRCA2* protonema cells (Fig. 5B and D, and Supplementary Fig. S6B). *AtBRCA2A* showed a weak RAD51 signal in 10% of cells, while *AtBRCA2A^ΔDBD^*showed only a diffuse nuclear signal in some cells (Fig. 5B and D, and Supplementary Fig. S6B). This diffuse signal in the *AtBRCA2^ΔDBD^* line may reflect the nuclear accumulation of RAD51. Thus, both PpBRCA2* and CpBRCA2 functionally mediated RAD51 recruitment after DSB formation, while AtBRCA2A with or without DBD failed to do so. Y2H assays confirmed *AtBRCA2A* interaction with *PpRAD51-2*, ruling out a lack of Arabidopsis-moss protein interaction (Supplementary Fig. S10). RT-PCR further confirmed the expression of PpBRCA2*, *CpBRCA2*, *AtBRCA2A*, and *AtBRCA2A^ΔDBD^* by detecting their respective transcripts (Supplementary Fig. S6C and D). These findings demonstrate that the canonical *AtBRCA2A* expressed under the *PpBRCA2* promoter is unable to complement HDR functions in moss, supporting differential evolutionary adaptation between canonical and noncanonical BRCA2 homologs.

### PpBRCA2 interacts with RAD51 through its BRC repeats

**Figure 6.**
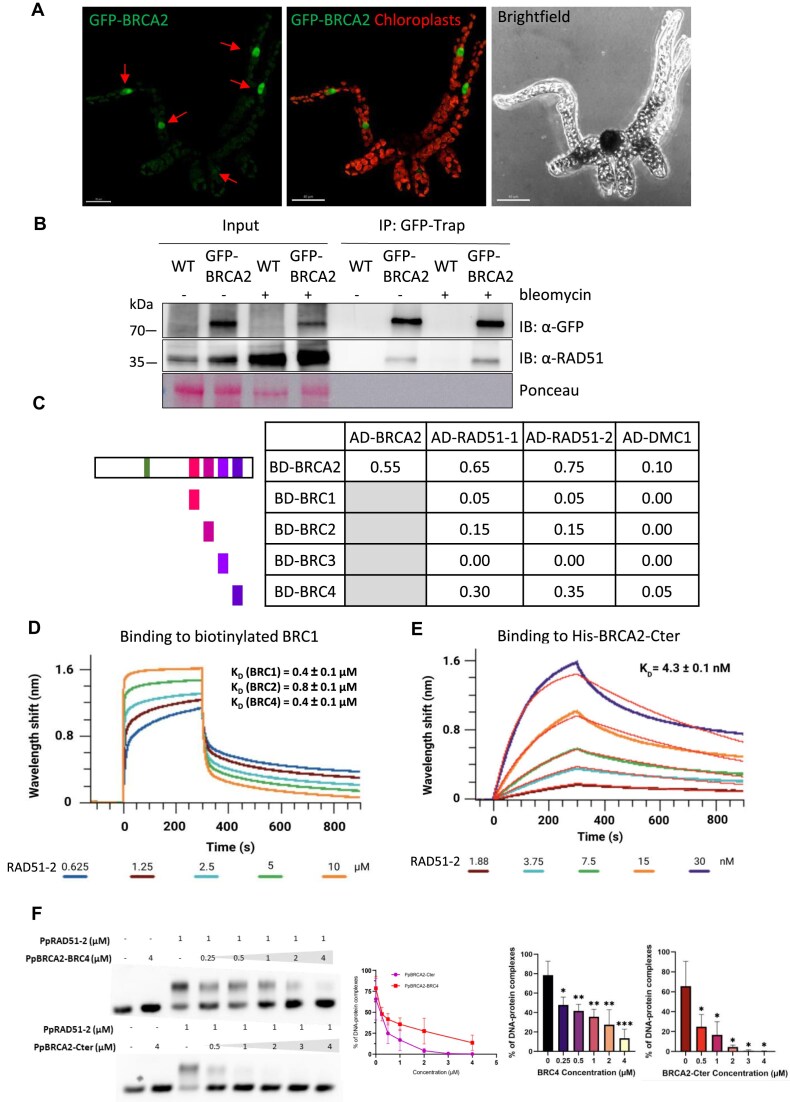
Cellular localization and interactions of PpBRCA2 protein (**A**) Representative image of a 1-week-old germinated GFP-BRCA2 spore. Red arrows indicate the strong GFP-BRCA2 signal in the nucleus. Chloroplasts’ autofluorescence is detected in red and light green. Scale bar: 30 μM. (**B**) CoIP of GFP-BRCA2 and PpRAD51. Total protein extracts of protonema from WT or GFP-BRCA2 lines treated with or without bleomycin were immunoprecipitated with GFP-trap beads. Input and immunoprecipitated fractions were detected on the same blot, which was divided into two parts for probing with anti-GFP and anti-RAD51 antibodies by western blotting. Ponceau staining is used as a loading control. (**C**) The interaction of full-length PpBRCA2 and four moss BRC repeats with RAD51-1, RAD51-2, and DMC1 was evaluated using Y2H assays. Each diploid was subjected to five-fold serial dilutions and spotted on SD/-LWH and SD/-LWHA media, where growing spots were assigned scores of 0.05 and 0.1, respectively. The final score for each diploid was obtained by summing the individual scores. A score of 0.75 shows the strongest interaction. Interactions not tested are marked in gray. BD; DNA-binding domain, AD; activating domain. (**D**) BLI interaction assays between biotinylated PpBRCA2-BRC peptides and PpRAD51-2. The BLI curves show PpBRCA2-BRC1 binding kinetics at increasing concentrations of PpRAD51-2 (0.625–10 μM). The Kd was calculated by fitting the plateau shift of the association curve (steady-state mode). The Kd affinity constants are presented for BRC1, BRC2, and BRC4 interactions. Experiments with replicates from PpBRCA2-BRC2, -BRC3, and -BRC4 are displayed in Supplementary Figs S11 and S12. No binding was observed with PpBRCA2-BRC3 (Supplementary Figs S11 and S12). (**E**) BLI interaction assays between 6His-PpBRCA2-Cter and PpRAD51-2. The BLI curves show PpBRCA2-Cter binding kinetics at increasing concentrations of PpRAD51-2 (1.88–30 nM), with replicates are presented in Supplementary Fig. S12A. (**F**) EMSA assays indicate an inhibitory effect of PpBRCA2-Cter or PpBRCA2-BRC4 on RAD51 filament formation. The 3 μM ssDNA (100 nt) was incubated with 1 μM RAD51-2 and increasing concentrations (0–4 μM) of PpBRCA2-Cter or PpBRCA2-BRC4 for 15 min at 37°C followed by migration of agarose gel. Quantification of RAD51-2-ssDNA complexes in the presence of PpBRCA2-Cter or PpBRCA-BRC4. The mean of four independent experiments is plotted with error bar indicating the standard deviation for each point. Histograms show reduction in filament formation with increasing concentrations of BRC4 and BRCA2-Cter.

We next generated a *P. patens* line expressing GFP fused at the N-terminus of PpBRCA2 by inserting the *eGFP* sequence upstream of the *BRCA2* start codon (Supplementary Fig. S1B). We determined the functionality of GFP-BRCA2 by analyzing plant growth, spore viability, meiosis, sensibility to bleomycin, HDR-dependent GT, and RAD51 focus formation. GFP-BRCA2 plants showed WT-like growth (Supplementary Fig. S7A and B) and meiosis, with well-shaped spores (Supplementary Fig. S7A) and a WT distribution of chromosome segregation (Supplementary Fig. S7D). However, the spore viability was slightly reduced, suggesting a minor functional defect (Supplementary Fig. S7C). Live imaging of germinated GFP-BRCA2 spores showed a nuclear GFP signal in some cells (Fig. 6A). This suggests that GFP-BRCA2 is detected as a nuclear protein in cells, which may be undergoing a cellular division, given its role in protecting stalled replication forks. Importantly, GFP-BRCA2 plants exhibited no increased sensitivity to bleomycin (Supplementary Fig. S7E and F) and maintained an HDR-dependent GT efficiency at 14.4%, close to the WT levels, with 98% of cassette integration occurring at the targeted locus (Supplementary Fig. S7I and J). Only a slight increase in RAD51-positive cells was observed after bleomycin treatment (Supplementary Fig. S7G). Altogether, our analysis shows that GFP-BRCA2 is functional for DNA repair and HDR.

We then assessed whether the expression of GFP-BRCA2 was induced in response to DSB formation, similar to RAD51. Western blot analysis revealed that GFP-BRCA2 protein steady-state levels did not increase to the same extent as PpRAD51 (Supplementary Fig. S7H) following DSB formation. Given that our AlphaFold3 analyses predicted high-confidence interactions for BRC repeats (except BRC3) with PpRAD51, we tested these interactions using various approaches. CoIP using GFP-trap beads confirmed a strong interaction between GFP-BRCA2 and RAD51 in moss independent of DSB formation (Fig. 6B). We further analyzed PpBRCA2 self-association and interaction with two *P. patens* RAD51 homologs (PpRAD51-1 and PpRAD51-2) by Y2H. Y2H assays detected a strong self-interaction of PpBRCA2 (Fig. 6C and Supplementary Fig. S10), consistent with BRCA2 oligomerization in humans [72, 73]. Additionally, the full-length PpBRCA2 strongly interacted with both PpRAD51 homologs (Fig. 6C and Supplementary Fig. S10). AlphaFold3 predicted that the PpBRCA2 BRC repeats do not interact with PpDMC1 (Supplementary Fig. S9A and S9C). Y2H assays consistently showed only weak interaction with PpDMC1 (Fig. 6C and Supplementary Fig. S10). We then tested each BRC repeat individually against PpRAD51-1, PpRAD51-2, and PpDMC1 in Y2H assays. Overall, isolated BRC repeats showed weaker interactions compared to the full-length PpBRCA2 (Fig. 6C and Supplementary Fig. S10). Among all BRCs, BRC4 displayed the strongest binding to both PpRAD51 proteins but a weak binding to PpDMC1 (Fig. 6C and Supplementary Fig. S10). BRC1 and BRC2 exhibited moderate interactions with PpRAD51 but no detectable interaction with PpDMC1, while BRC3 failed to interact with both recombinases (Fig. 6C and Supplementary Fig. S10), confirming AF predictions (Fig. 1C).

We next calculated the binding affinities to validate Y2H results using BLI assays with purified PpRAD51-2, PpDMC1 proteins (Supplementary Fig. S8), and BRC peptides. Biotinylated BRC peptides immobilized on a sensor were incubated with increasing concentrations of recombinant PpRAD51-2 or PpDMC1, followed by buffer immersion to monitor the association and dissociation kinetics (Kd). We measured Kd values of 0.4, 0.8, and 0.4 μM for PpRAD51-2 interactions with BRC1, BRC2, and BRC4, respectively (Fig. 6D, and Supplementary Figs S11A and C and S12A). No detectable interaction was recorded between BRC3 and PpRAD51-2 (Supplementary Figs S11B and S12A) or between BRC repeats and PpDMC1 (Supplementary Fig. S9B). Sequence alignments revealed a glycine-to-alanine substitution after the conserved FxxA motif in PpBRCA2-BRC3, potentially explaining its lack of interaction with PpRAD51. Replacing alanine with glycine partially restored PpRAD51 binding (Supplementary Fig. S11D), confirming the importance of the glycine residue. PpBRCA2-BRC1, BRC2, and BRC4 also exhibit a leucine at position 3 of the FxxA motif, where a serine or threonine residue is present in human BRC repeats, playing an important role in the structure of the BRCA2-RAD51 interface [65]. Finally, all four moss BRC repeats have a phenylalanine at position 9 close to this leucine, instead of an aliphatic hydrophobic residue in humans. Thus, even if the BRCA2-RAD51 interface is remarkably conserved between moss and human, a few mutations might characterize the moss binding interface. We finally assessed the collective contribution of the BRC repeats using purified recombinant PpBRCA2-Cter, which contains all four BRCs. BLI assays showed a 100-fold stronger affinity of PpBRCA2-Cter for PpRAD51-2 (Kd = 4.3 nM) compared to individual BRC repeats (Fig. 6E and Supplementary Fig. S12A), highlighting a cooperative binding effect among BRCs.

Structural studies of the human BRCA2 BRC4-RAD51 complex suggest that BRCA2 recruits monomeric RAD51 at DSB sites via its FxxA motifs [65]. Excess human BRC4 peptides can disassemble preformed RAD51 filaments on ssDNA [74, 75]. However, a ternary complex of truncated human BRCA2 containing all eight BRC repeats with RAD51, and ssDNA filaments is observed in the presence of nonhydrolyzable AMP-PNP [76]. We determined the effects of PpBRCA2-BRC4 and PpBRCA2-Cter on the assembly of PpRAD51-ssDNA filaments by EMSA to unravel how PpBRCA2 regulates RAD51-ssDNA filaments. EMSA analysis showed that both PpBRCA2-Cter and BRC4 prevent the PpRAD51-ssDNA filaments formation in a concentration-dependent manner (Fig. 6F). However, complete filament inhibition required different peptide concentrations: 2 μM for PpBRCA2-Cter and >4 μM for PpBRCA2-BRC4, confirming a significantly higher affinity of PpBRCA2-Cter for PpRAD51-2. These results highlight the cooperative action of BRC repeats in regulating RAD51 filament assembly.

Taken together, PpBRCA2 plays a crucial role in HDR by mediating RAD51 recruitment through its BRC repeats. While individual BRCs show varying affinities, their combined action significantly enhances RAD51 binding, underscoring a conserved mechanism of BRCA2-mediated recombinase regulation. In contrast, PpBRCA2 exhibits minimal interaction with PpDMC1, suggesting a limited role in meiosis-specific recombination.

### PpBRCA2 binds to ssDNA via its N-terminal region

**Figure 7.**
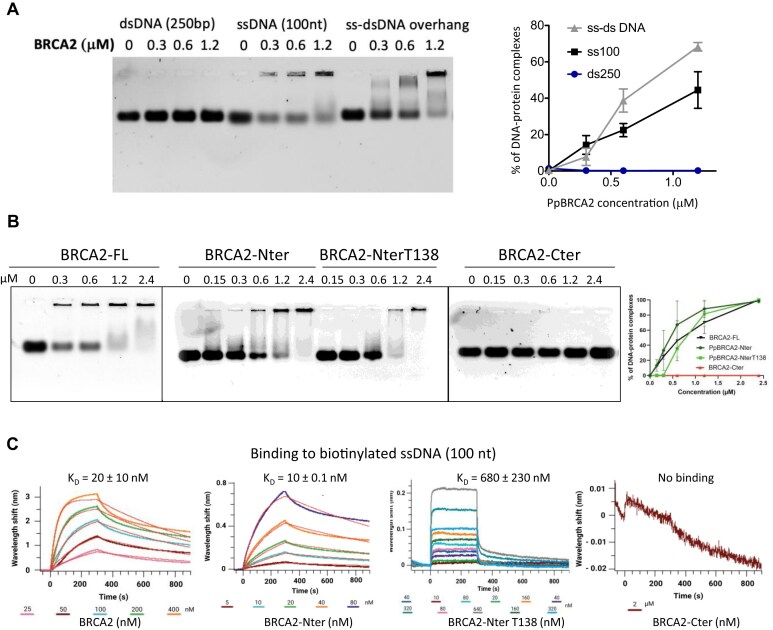
Interaction between PpBRCA2 and DNA by EMSA and BLI. (**A**) EMSA was performed by incubating 2 μM Cy5-labeled dsDNA (250 bp), ssDNA (100 nt), or ss–dsDNA (150 nt–100 bp) overhang substrates with increasing PpBRCA2 concentration (0–2.4 μM) for 7 min at 37°C followed by migration on agarose gel. The quantification of DNA–protein complexes showed that PpBRCA2 efficiently binds to ssDNA and ss–dsDNA overhang, but not to dsDNA. (**B**) The interaction of N-terminal and C-terminal regions of PpBRCA2 with ssDNA was assessed by EMSA. The increasing concentration of PpBRCA2, PpBRCA2-Nter, PpBRCA2-NterT138, or BRCA2-Cter (0–2.4 μM) was incubated with 2 μM ssDNA (100 nt) for 7 min at 37°C followed by migration on agarose gel. The PpBRCA2 and PpBRCA2-N-ter and PpBRCA2-NterT138 bind to ssDNA, but no binding was observed with PpBRCA2-Cter. (**C**) BLI assays were performed to measure the interaction between biotinylated ssDNA (100 nt) and PpBRCA2, PpBRCA2-Nter, PpBRCA2-NterT138, and PpBRCA2-Cter. The Kd values as a function of protein concentrations are obtained by fitting both the association and dissociation curves with replicates presented in Supplementary Fig. S12. The shape of BLI curves and Kd constants together indicate that multiple sites at the BRCA2 N-terminal can interact with ssDNA.

Human BRCA2 binds to ssDNA through its canonical DBD as well as via its disordered N- and C-terminal regions [34, 35, 65]. We explored whether the disordered PpBRCA2 exhibits similar binding with various DNA substrates. We used purified recombinant PpBRCA2, PpBRCA2-Nter, and PpBRCA2-Cter to perform EMSA assays. Our EMSA analysis revealed that full-length PpBRCA2 binds to a 100 nt ssDNA and 150 nt–100 bp ss–dsDNA overhang but does not associate with 250 bp dsDNA (Fig. 7A). Further analysis identified that the N-terminal region of PpBRCA2 mediates ssDNA binding (Fig. 7B). To quantify binding affinities, we performed BLI analyses using biotinylated ssDNA immobilized on a sensor with increasing concentrations of recombinant PpBRCA2, PpBRCA2-Nter and PpBRCA2-Cter. PpBRCA2 and PpBRCA2-Nter showed strong ssDNA binding with dissociation constants (Kd) of 10–20 nM (Fig. 7C and Supplementary Fig. S12B). In contrast, PpBRCA2-Cter displayed no detectable binding to ssDNA, consistent with the EMSA results (Fig. 7C and Supplementary Fig. S12C). These findings demonstrate that the noncanonical PpBRCA2 binds to ssDNA through its N-terminal region. To delimitate the PpBRCA2 ssDNA binding region, we searched for positively charged regions conserved between *P. patens* and *C. purpureus*. We identified two regions that are conserved in PpBRCA2-Nter and CpBRCA2: region 1–138 (PpBRCA2-NterT138; 24 positively charged residues including the NLS) and region 175–220 (eight positively charged residues). We produced recombinant PpBRCA2-NterT138 protein and tested its binding to ssDNA. PpBRCA2-NterT138 showed about 50 times weaker affinity for ssDNA compared to PpBRCA2-Nter (Fig. 7B). Thus, PpBRCA2 binding to ssDNA might rely on multiple positively charged patches distributed along the highly positively charged PpBRCA2-Nter.

## Discussion

We identified and characterized a unique plant BRCA2 homolog from *P. patens* (PpBRCA2), which has conserved BRC repeats but lacks the canonical DBD. Our study supports the existence of two types of BRCA2 homologs (Fig. 1E): (i) a canonical BRCA2, which includes a well-conserved folded DBD in most organisms, and (ii) a noncanonical BRCA2, which lacks the canonical DBD as found in drosophila [2, 4] and mosses. *Physcomitrium patens brca2Δ* mutants share similarities with canonical *brca2* plant mutants but also exhibit distinct phenotypes. While BRCA2 loss in mammals is embryonic lethal [77], *brca2Δ* mutants are viable in plants [3, 7] and drosophila [2, 4]. *Physcomitrium patens brca2Δ* mutants exhibit slow growth, with a ∼30% reduction in plant size, in contrast to the apparent normal growth observed in *A. thaliana brca2* [7]. We provide compelling evidence that PpBRCA2 promotes DSB repair in somatic and meiotic cells. PpBRCA2 plays a critical role in DSB repair and genome stability by preventing hypersensitivity against genotoxic agents and limiting spontaneous mutation occurring in the genome, consistent with *P. patens rad51-1rad51-2* phenotypes [61] and the elevated mutation frequency observed in cells isolated from canonical *BRCA2-*mutated patients in humans [78]. The noncanonical PpBRCA2 acts in somatic HDR as *brca2Δ* mutants exhibit a 10-fold reduction in GT, accompanied by an increase in random integrations. Further, the absence of RAD51 focus formation at DSBs in *P. patens brca2Δ* supports the role of PpBRCA2 as a RAD51 mediator. Loss of PpBRCA2 may either prevent RAD51 recruitment at DSB sites or lead to active RAD51 filament dissociation by antirecombinase proteins such as FIGL1 [62]. This suggests that PpBRCA2 is essential for the nucleation of RAD51 filaments.

Both *A. thaliana* and rice *brca2* mutants produce nonviable pollens and meiocytes fail to form bivalents owning to unrepaired breaks, leading to chromosome fragmentation detected from metaphase I onwards stages [3, 7]. Similarly, *P. patens brca2Δ* mutants produce mostly nonviable spores and exhibit severe meiotic repair defects. Surprisingly, milder meiotic defects were observed at metaphase I in *P. patens brca2Δ* meiocytes. Only a small fraction of *P. patens brca2Δ* metaphase I cells displayed the bivalent shortage with a mix of univalent and bivalents, the remaining showed WT levels of bivalents. The *P. patens rad51-1rad51-2* meiocytes also displayed similar milder defects in bivalent formation with most cells having WT levels. However, a strong meiotic repair defect in terms of chromosome bridges and fragmentation was detected at anaphase I and subsequent stages. This indicates that BRCA2 and RAD51 are not essential for bivalent formation in moss, unlike their counterparts in Arabidopsis and rice. One speculation is that PpDMC1 can promote bivalents independent of BRCA2 and RAD51, though further work is necessary to confirm this hypothesis. Nonetheless, nonviable spores and chromosome breakage detected after metaphase I suggest critical roles of PpBRCA2 in RAD51-dependent meiotic DSB repair. Overall, the noncanonical PpBRCA2 mediates both somatic and meiotic RAD51 functions.

Canonical BRCA2 mediates HDR through its direct physical interaction with both RAD51 and DMC1 recombinases. We found a robust interaction between full-length PpBRCA2 and PpRAD51 homologs, with the BRC repeats binding to PpRAD51 at varying affinities. Isolated BRC1, 2, and 4 repeats showed micromolar affinities for RAD51, while BRC3 did not bind due to a glycine-to-alanine mutation following the FxxA motif. These findings are in concordance with previous studies showing varying interaction affinities of BRC repeats [30, 63]. PpBRCA2-Cter (with all BRC repeats) displayed a 100-fold increase in binding affinity, reaching the nanomolar range, indicating a strong synergistic effect of the four BRCs on RAD51 binding. Our BLI data combined with the AF3 modeling suggest that PpBRCA2 BRC1, BRC2, and BRC4 repeats bind to monomeric RAD51-2, similar to human BRC4 repeat [65]. Furthermore, PpBRCA2-Cter and BRC4 in higher concentrations impede RAD51-ssDNA filament formation, suggesting an inhibitory effect as previously observed with human BRC repeats [74, 75]. Altogether, our data indicate that canonical and noncanonical BRCA2 orthologs regulate RAD51 loading through similar mechanisms.

Unlike canonical BRCA2 that strongly interacts with DMC1 [31, 79], PpBRCA2 BRC repeats bind to PpDMC1 with much lower affinity than to PpRAD51, supported by the low interaction scores from AF modeling. Despite a residual interaction in Y2H assays, no interaction with PpDMC1 was detected using in vitro assays. We propose that PpBRCA2 may have a conserved mediator function through RAD51 interaction in both somatic and meiotic cells and a less important role in DMC1-mediated recombination.

BRCA2 orthologs exist across eukaryotes, but their evolutionary dynamics across species remain puzzling, partly due to high sequence variability outside the BRC repeats and canonical DBD [38]. The evolutionary origin of noncanonical BRCA2 homologs is particularly unresolved. One hypothesis is that they arose through the loss of the canonical DBD in certain lineages. Our analysis revealed that DBD loss is common to three moss genera—*P. patens*, *C. purpureus*, and *F. hygrometrica*. Although limited number of annotated moss genomes restrict broader analysis, the DBD loss likely occurred in the moss common ancestor after diverging from liverworts and hornworts. We propose that moss-specific evolutionary events led to the emergence of noncanonical BRCA2 within the bryophytes. Our data also suggest distinct evolutionary trajectories for canonical and noncanonical BRCA2 homologs. *CpBRCA2* can functionally complement *brca2Δ-1*, whereas AtBRCA2 cannot, despite interaction with PpRAD51-2, indicating functional divergence. We propose that the HDR machinery differs in moss and angiosperms. Moreover, interactions involving the DBD, such as with DSS1, may not be essential for noncanonical BRCA2 functions. While DSS1 is crucial for canonical BRCA2 function [26], it appears dispensable in drosophila, where no genetic or protein interactions between drosophila BRCA2 and DSS1 have been observed [2]. Interestingly, DSS1 can also coordinate DNA repair via interaction with RAD52 [27], a protein functionally related to BRCA2. The role of the two DSS1 homologs in *P. patens* remains to be explored in the context of HDR in the future.

Canonical BRCA2 homologs contain additional DNA-binding regions aside from the well-conserved and folded DBD. Human BRCA2 contains two such auxiliary regions at its N-terminal Domain (NTD) and C-terminal (CTRB) [29, 34, 35]. While the canonical DBD provides specificity for ssDNA binding, the auxiliary domains can bind both ssDNA and dsDNA [34, 35, 37]. The current model suggests that human BRCA2 recruits RAD51 by either directly binding ssDNA through its canonical DBD or sliding on dsDNA via the auxiliary domains [37]. The high frequency of pathogenic BRCA2 mutations within the canonical DBD in cancer patients underscores the importance of ssDNA binding of BRCA2 *in vivo* [38, 39]. However, deleting canonical DBD in mammalian BRCA2 does not cause lethality or completely disrupt HDR and RAD51 recruitment at DSBs, suggesting the auxiliary domains may compensate [80–83]. Alternatively, RAD52 could potentially promote HDR when BRCA2 is downregulated. The presence of noncanonical BRCA2 homologs in drosophila and mosses suggests that the canonical DBD may not be essential for HDR in eukaryotes. But how do noncanonical BRCA2 homologs with no DBDs facilitate RAD51 focus formation? We demonstrate that the noncanonical PpBRCA2 interacts with RAD51 through BRC repeats and can bind specifically to ssDNA via its N-terminal disordered region outside the conserved BRC repeats. We speculate that noncanonical PpBRCA2 promotes exchange between RPA and RAD51 on ssDNA at the DSB sites through its direct binding. This reinforces the idea that the DNA binding activity, rather than the presence of canonical DBD, is likely a conserved feature among BRCA2 orthologs throughout evolution.

## Supplementary Material

gkaf856_Supplemental_Files

## Data Availability

All datasets reported in this work are available from the corresponding authors on request.
